# Protocol for high-resolution 3D visualization of insect regenerating legs through micro-computed tomography

**DOI:** 10.1016/j.xpro.2024.103342

**Published:** 2024-09-26

**Authors:** Liang Li, Kai Cheng, Jiru Zhong, Shaojuan Zheng, Chenjing Zhao, Yejie Wen, Sheng Li, Chonghua Ren

**Affiliations:** 1 Guangdong Provincial Key Laboratory of Insect Developmental Biology and Applied Technology, Guangzhou Key Laboratory of Insect Development Regulation and Application Research, Institute of Insect Science and Technology, School of Life Sciences, South China Normal University, Guangzhou 510631, China; 2 Guangdong Laboratory for Lingnan Modern Agriculture, Guangzhou 510631, China; 3 Guangmeiyuan R&D Center, Guangdong Provincial Key Laboratory of Insect Developmental Biology and Applied Technology, South China Normal University, Meizhou 514779, China; 4 Department of Biology, Taiyuan Normal University, Jinzhong 030619, China

**Keywords:** Cell Biology, Developmental biology, Evolutionary biology, Microscopy, Molecular Biology

## Abstract

Appendage regeneration occurs within the opaque exoskeleton in arthropods, making it challenging to visualize the regenerative processes dynamically. In this protocol, we present a strategy to scan and capture the high-resolution details of microstructural tissues at certain regeneration points through micro-computed tomography (micro-CT). We describe steps for tissue preparation, fixation, critical point drying, micro-CT scanning, and 3D visualization. This approach promises significant utility in the field of regeneration, particularly in studies involving arthropods such as insects and crustaceans.

For complete details on the use and execution of this protocol, please refer to Ren et al.^1^

## Highlights

• Steps for collecting insect regenerating legs
• Instructions for micro-CT sample pre-processing
• Procedure to obtain the 3D structure of regenerating legs

## Abstract (editor highlights)

Publisher’s note: Undertaking any experimental protocol requires adherence to local institutional guidelines for laboratory safety and ethics.

## Graphical abstract

**Figure:**
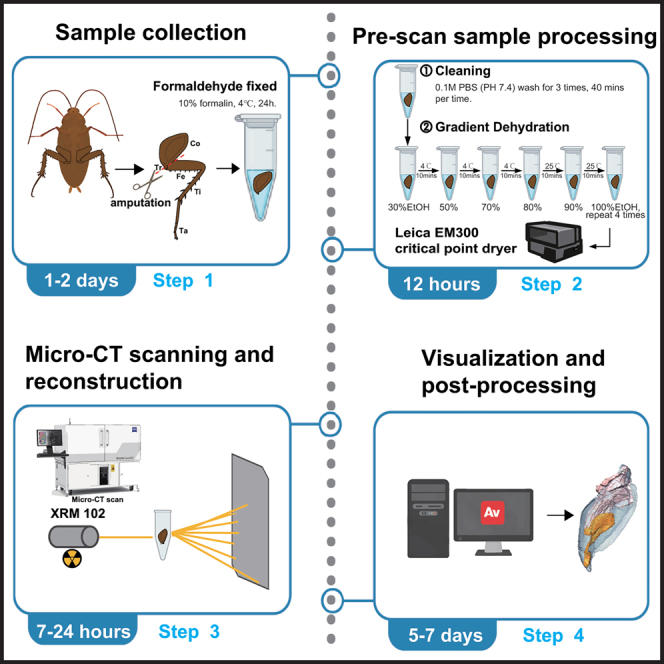

## Before you begin

1. Insect preparation for leg regeneration study.
  a. Anesthetize the nymphal insects (e.g., American cockroach, *Periplaneta americana*) using CO_2_.
  b. Excise the distal tarsus, tibia, and femur of a metathoracic hindleg at the trochanter-femur junction, preserving the trochanter and coxa for regeneration observation.
  c. Allow approximately 10 days for nymphs to undergo regeneration at 27°C with a relative humidity of 70%–80% in plastic cages.
2. Prepare fixation and dehydration reagents as outlined in the key resources table.
3. Ensure proper functionality of the micro-CT apparatus by activating the machine and verifying optimal settings.
4. Acquire proficiency in operating the Amira software through basic training sessions.

### Institutional permissions

Experiments on insects received appropriate institutional approvals and were performed in accordance with institutional guidelines.

Prior to executing the protocol presented herein, investigators must also acquire relevant permissions from their institution and follow IACUC guidelines and regulations.

### Preparation of sample collection

**Timing: 10 min**

5. Perform the sample collection with the following materials:
  a. Vannas Spring Scissors (8 cm).
  b. Stereoscopic microscope.
  c. CO_2_ tank.
  d. 1.5 mL Eppendorf tube.
6. Open the valve of the CO_2_ tank, anesthetize the nymphs via carbon dioxide.

***Note:*** Ensure all American cockroach nymphs are maintained under specific conditions, including housing in a dedicated animal facility with a 12/12-h light/dark cycle and access to food and water *ad libitum*.

### Pre-scan sample processing preparation

**Timing: 1 h**

7. Prepare 10% formalin for fixation alongside other requisite solutions for dehydration.
  a. 10% Hydrochloric Acid (HCl): Dilute 35%–37% hydrochloric acid to a 10% concentration using deionized water.
  b. 1× PBS: Prepare a 10-fold dilution from stock 10× PBS with deionized water.
  c. Ethanol solution: Prepare 90%, 80%, 70%, 50%, and 30% v/v solution of ethanol in deionized water.
8. Cut the filter paper into 1.2 cm × 3 cm strips with scissors.
9. Ensure adequate sample baskets, parts of the critical point dryer, are available and wash them thoroughly with ethanol.

***Note:*** Tissue fixation and critical point drying are required for the investigation of insect leg regeneration.

***Note:*** When handling acidic solutions, adhere to standard laboratory safety protocols, including the use of gloves and safety glasses to prevent contact with mucous membranes.

***Alternatives:*** Any fixative or formaldehyde solutions may be employed for sample fixation, with necessary adjustments in concentrations. (e.g., 4% formaldehyde, 10% formalin).

## Key resources table

**Table:** 

REAGENT or RESOURCE	SOURCE	IDENTIFIER
**Chemicals, peptides, and recombinant proteins**		

Formalin	Sigma-Aldrich	F8775
Sodium phosphate dibasic dodecahydrate (Na_2_HPO_4·_12H_2_O)	Macklin	S818118
Potassium phosphate monobasic (KH_2_PO_4_)	Macklin	P815662
Potassium chloride (KCl)	Macklin	P816347
Sodium chloride (NaCl)	Sigma-Aldrich	S9888
Hydrochloric acid	Sigma-Aldrich	258148
Ethanol	Sigma-Aldrich	E7023

**Experimental models: Organisms/strains**		

*Periplaneta americana*	N/A	N/A

**Software and algorithms**		

Amira 6.0.1	Thermo Fisher Scientific	www.thermofisher.com
NRecon	Bruker	N/A
DataViewer	Bruker	N/A

**Other**		

CO_2_ tank	N/A	N/A
Micro-CT system	Bruker	SKYSCAN 2214
Critical point dryer	Leica	EM ACE600
1.5 mL Eppendorf tubes 3810×	Eppendorf Innovation Company	www.eppendorf.com
Vannas Spring scissors (8 cm)	RWD Life Science	Cat#S11001-08
Microscope	Olympus Corporation	SZ61

***Note:*** There are other Micro-CT systems can be selected such as Xradia Context micro-CT from ZEISS.

***Note:*** There are other Critical point dryer instruments can be selected such as EM CPD300 from Leica.

## Materials and equipment

**Table:** 10 × PBS (1 L)

Reagent	Final concentration	Amount
NaCl	1.37 M	80.062 g
KCl	0.027 M	2.012 g
KH_2_PO_4_	0.018 M	2.449 g
Na_2_HPO_4·_12H_2_O	0.1 M	35.814 g
Deionized water	-	1 L

***Note:*** Once high temperature sterilized, store at 4°C for 6 months.

### Micro-CT settings

• Micro-CT scanning was conducted at the South China Botanical Garden utilizing SKYSCAN 2214 Micro-CT scanners. Materials like polypropylene exhibit low X-ray absorption, rendering 1.5 mL Eppendorf tubes optimal as sample holders. Parameters such as X-ray Voltage (kV), Current (μA), Exposure Time (ms), and Filter Setting were pre-optimized to ensure ample transmission through the samples. Resolution, scan orbit, rotation step, and final scanning duration were tailored according to the specific sample characteristics. Detailed parameters for the showcased samples are delineated in Table 1.

**Table 1:** Representative micro-CT Settings for Presented Samples

Settings	
Micro-CT instrument	SKYSCAN 2214
X-ray source	<0.5 microns spot size, energy range 20–160 kV
Power	30 kV/130 μA
Filter	None
Resolution (pixel size)	3 μm
Object to Source	26.971 mm
Camera to Source	315.558 mm
Camera binning	2 × 2
Scan Orbit	360 degrees
Rotation Step	0.2 degrees
Exposure time	1300 ms or variable upon certain conditions

***Note:*** Considering the design and protective measures of the micro-CT apparatus, radiation safeguards like a lead apron or badge are typically unnecessary while operating the machine. However, users are advised to consult their institutional safety board and/or radiation safety officer for verification.

### Amira software specifications

• Amira, developed by Thermo Scientific, stands as an advanced image analysis software tool. Primarily a 3D visualization software, Amira can be used in analytical tasks within the medical domain and a diverse array of other fields such as life sciences, materials sciences, cellular studies, and neurosciences. With robust segmentation and image processing capabilities, Amira empowers life science and biomedical researchers to derive qualitative and quantitative data across various scales and modalities.
• This software facilitates both manual and automated tools for data segmentation, offering impactful animations and high-quality images suitable for publication purposes.
• Users can leverage Amira to visualize, edit, and refine surfaces extracted from CT data, preparing them for 3D printing or digital dissemination.

***Note:*** There are other free software solutions for the segmentation, analysis and visualization of 3D CT data, such as Dragonfly (free academic license), 3D Slicer, SPIERS, tomviz, VTK etc.^2^

### Other software specifications

• NRecon (Bruker MicroCT) is employed for reconstructing raw micro-CT data.
• DataViewer (Bruker MicroCT) is utilized for 2D visualization of all 3 planar sections of reconstructed data.

***Note:*** There are other software solutions to choose from, such as DICOM converter, Fiji, and Micro DICOM Viewer.

## Step-by-step method details

### Sample collection

**Timing: 1–2 days**

This section provides stepwise instructions for sample collection.

**Figure 1:**
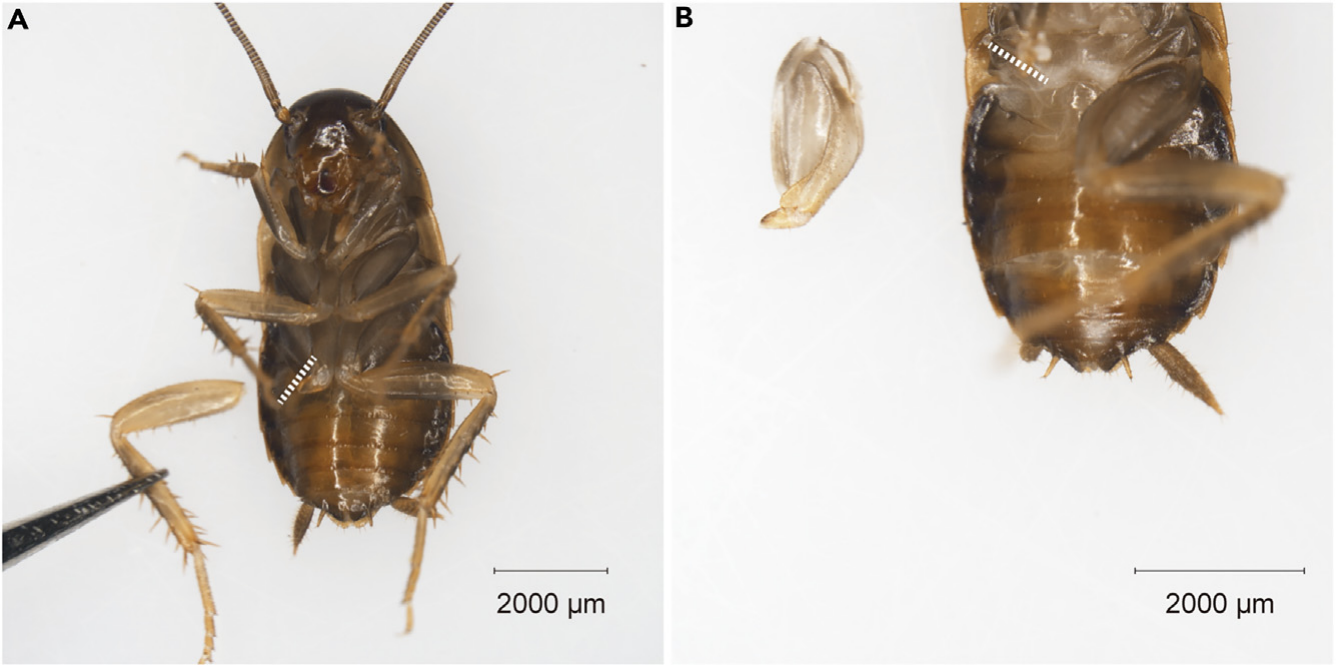
Amputation and sampling of *P. americana* regenerating legs (A) Gross photograph of the ventral side of *P. americana*. Legs are divided into five sections from the proximal to the distal: coxa, trochanter, femur, tibia, and tarsus. Amputation was performed between the trochanter and femur. The femur, tibia, and tarsus were removed. (B) The entire coxa and trochanter were excised from the proximal end when sampling. Scale bar = 2000 microns.

1. After CO_2_ anesthesia, cut the hindlegs of cockroaches at the trochanter-femur junction under a microscope with Vannas Spring Scissors (8 cm) (Figure 1A). Allow the cockroaches to live for several days (about 10 days in this case) to regenerate new legs.
2. Harvest the regenerating legs (coxa and trochanter) under a microscope with Vannas Spring Scissors (Figure 1B).
3. Transfer them to a 1.5 mL Eppendorf tube.
4. Add approximately 1 mL of 10% formalin to the Eppendorf tube.
5. Position the tube containing the fixative and sample on a shaker at 4°C for over 24 h. Troubleshooting 1.

***Note:*** Following CO_2_ anesthesia of the insects, it is advisable to place them on ice until the sampling procedure is finalized. About 30 min is fine for cockroach to stay on ice.

**CRITICAL:** It is important to ensure that the sample is fixed for more than 24 h. Insufficient fixation time might lead to samples damage.

### Pre-scan sample processing

**Timing: 12 h**

This section provides stepwise instructions for sample dehydration and for obtaining the sample that could be scanned directly from critical point drying.

6. Rinse the fixative with phosphate buffer solution (1× PBS, pH 7.4), thrice within 2 h, each rinse lasting 40 min. Store the sample at 4°C in a refrigerator.
7. Gradient Dehydration. Troubleshooting 1.
  a. Replace the PBS with 30% ethanol and place in a refrigerator at 4°C for 10 min.
  b. Replace with 50% ethanol and place in a refrigerator at 4°C for 10 min.
  c. Replace with 70% ethanol and place in a refrigerator at 4°C for 10 min.
  d. Replace with 80% ethanol at 25°C for 10 min.
  e. Replace with 90% ethanol at 25°C for 10 min.
  f. Replace with 100% ethanol at 25°C for 10 min.
  g. Repeat step f for four times.

***Note:*** Ensure that the solutions utilized in this section are adequate for complete submersion of the sample. The ethanol solutions should be replaced quickly to prevent the samples from drying out. The rotation is required for gradient dehydration steps.

***Note:*** It is advised to store samples at 4°C throughout processing to inhibit microbial reproduction.

8. Wrap each sample in a piece of filter paper and mark in pencil separately.
9. Transfer them to the sample basket, one sample per basket.
10. Position the baskets with samples inside the critical point dryer.
11. Turn on the critical point dryer (Table 2).

**Table 2:** Drying Programs for presented samples

Steps	Settings	
Cool	Temperature	10°C
CO_2_ IN	Speed	Slow
	Delay	200 s
Exchange	Speed	5
	Cycles	20
Heat	Temperature	40°C
	Speed	Slow
Gas OUT	Speed	slow 100%
12. Upon completion of 2 h drying process, retrieve the samples and store them in a vacuum dryer.

***Note:*** Avoid using oil pens to make marks on filter paper, oily notes can be dissolved by alcohol.

**Pause point:** Store samples in vacuum for up to 1 month or proceed to the next step.

### Micro-CT scanning and reconstruction

**Timing: 7–24 h**

This section provides stepwise instructions for setting up micro-CT parameters, and image reconstruction.

13. Prepare and position the sample inside a 1.5 mL Eppendorf tube. Noting that during the scanning, the sample needs to be fixed with an X-ray low absorption material to prevent sample movement, such as cotton.

***Note:*** If the 1.5 mL Eppendorf tube does not fit the sample, consider wrapping the sample in parafilm and securing it in a trimmed 50 mL conical tube.

14. Insert the Eppendorf tube containing the sample in the micro-CT holder. Align the tube symmetrically and perpendicular to the X-ray beam path to maintain consistent X-ray penetration depth throughout the scan rotation.
15. Define the optimal scanning parameters for your sample. Begin with acquiring bright field images. Input the scan parameters based on the requirements of your study.
  a. Scan Name.
  b. Rotation Step (deg).
  c. Total Rotation (180° vs. 360°).
  d. Resolution (pixel size).

***Note:*** The scanning duration for the sample will largely depend on the number of FOV sections required to capture the entire sample. A partial volume scan will decrease the scan time, but the sample must be centered.

**Figure 2:**
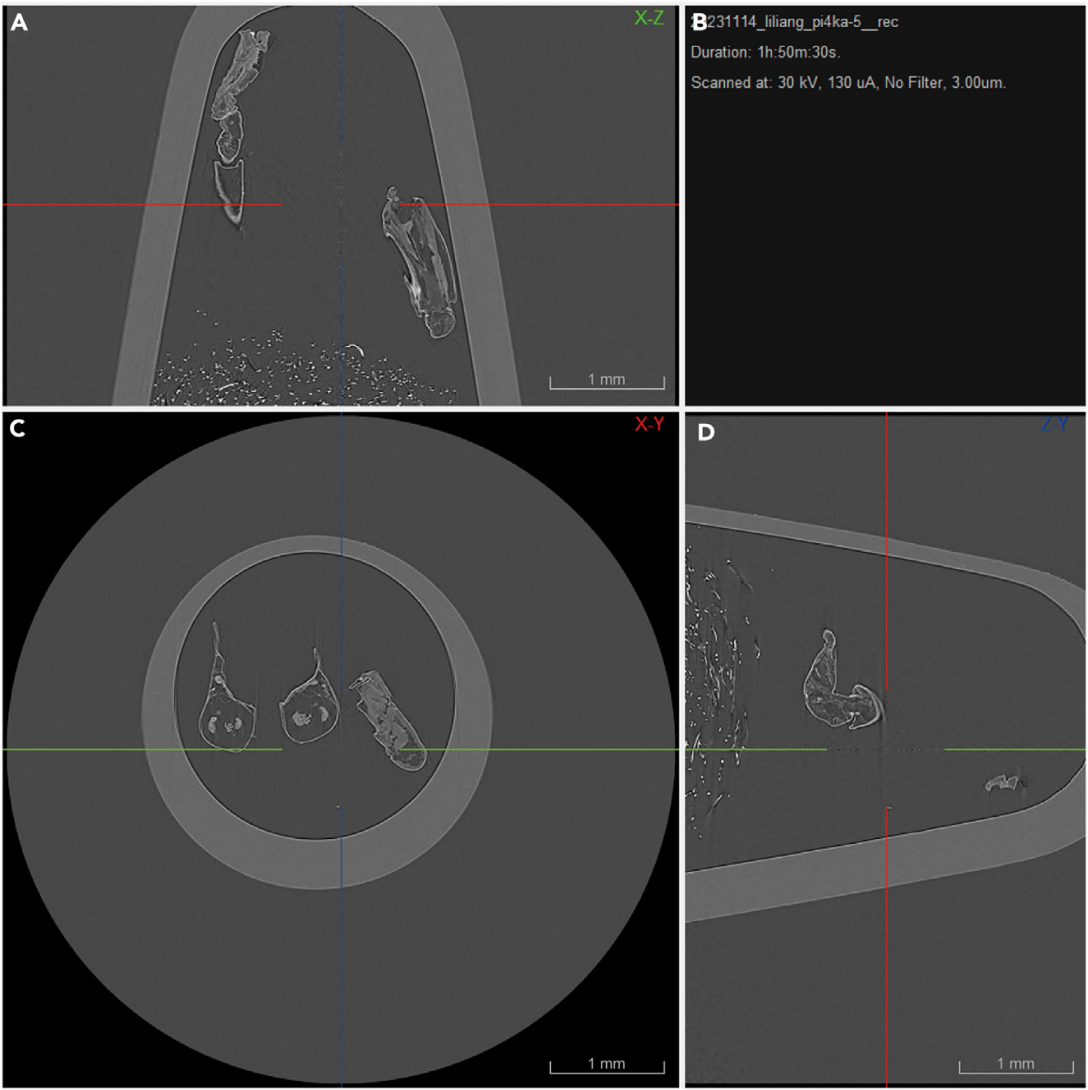
Three-panel view of 2D reconstruction in NRecon software The image displays a three-panel view within the NRecon software which enables the observation and interaction with 2D slice data across three distinct orientations: X-Y (A), X-Z (C), and Z-Y (D). (B) showcases the major information when scanning. Each panel exhibits the corresponding locations of 2D images in various planes with multicolor crosshairs for precise referencing and analysis. Scale bar = 1000 microns.

***Note:*** Maintain consistent parameters across samples, encompassing beam hardening correction, dynamic range, and smoothing settings. Reconstruction duration is influenced by scan section quantity, image resolution, and computational capabilities.

***Note:*** Save reconstructed data without compression or down-sampling (e.g., as 16-bit TIFF or BMP files). In cases of limited storage or data sharing requirements, consider compressed formats like 8-bit PNG or 8-bit TIFF.

16. Turn on the X-ray source and commence the scanning process.
17. Upon completion of the scan, return the sample to the vacuum dryer.
18. Reconstruction of raw data and preliminary visualization (Figure 2).
  a. Launch images in NRecon software.
  b. Implement ring artifact reduction and beam hardening correction as necessary.
  c. Refine the alignment for each scan section.
  d. Start the reconstruction.

***Optional:*** Utilize DataViewer (Bruker software) to rotate the reconstructed images if desired and export the newly adjusted reconstruction.

**Pause point:** The samples can be restored in vacuum condition for reusing. The data can be used for further analysis.

### Visualization and post-processing

**Timing: 5–7 days**

This section provides stepwise instructions for processing acquired 3D image data utilizing the Amira software. The following process is used as an example by scanning one sample at a time.

19. To import all reconstructed images into the Amira software, select **Open Data.**
  a. Choose all required .tiff or .bmp files.
    ***Note:*** After that, a window with the title Image Read Parameters will open.
  b. Set the voxel size in **Resolution** using the information on the header .log file.
  c. Press **OK.**
    ***Note:*** If the data size is large, a window with the title Out-of-Core Data will open (before the Image Read Parameters box opens) querying how to read the data. Select **Read complete volume into memory**. Press **OK**.
20. In **Main Panel > Project**, a new object is created with the name of the .bmp.
  a. Click on this object.
  b. Change the object name to the desired text (e.g., raw.am).

***Optional:*** Click on raw.am and click **File > Save Data**.

21. Right-click raw.am object, select the option **Ortho Slice > Create** to see the reconstructed 2D image of samples. Troubleshooting 2 and 3.

***Note:*** Slide ▲ of **Slice Number** in **Properties** to preview different slices.

***Optional:*** Right-click raw.am object and select the option **Volren > Create** for 3D visualization of the sample.

***Note:*** Amira models might left/right mirrored with respect to the original sample when using Bruker data. If data is mirrored, right-click raw.am object and select the option **Crop Editor**. A window with the title **Help** will open and then choose **flip z** to inverse the Z-axis.

***Note:*** If it's hard to see the boundaries of the sample structure, right-click raw.am object and select the option **Brightness/Contrast**. Set contrast value in **Parameters**, usually between 1 and 3. Click **Apply**. The subsequent label operation is performed on the raw-filtered∗ object.

***Alternatives:*** Colormap sliders in the **Ortho Slice** can be used to change the mapping of the grayscale values onto the colormap, thereby allowing to change the visual representation of the image data.

***Note:*** Unsaved object names are suffixed with ∗.

22. Right-click raw.am object, select the option **Edit New Label Field > Create**, and you’ll be directed to **Segmentation** (Figure 3A).
  ***Note:*** If you go back to **Project**, you will see that a new raw.labels∗ object has appeared, stuck to the bottom of raw.am object.
  a. Select **New** to create a new material and assign it a name and color (Figure 3B).
  b. Locate your material on the right screen interface. Label it by coloring it in with brush or lasso tool (Figure 3C).
    ***Note:*** On the top left panel (**Materials**), you will see two existing materials (Exterior and Inside). Ignore these two.
    ***Note:*** You can use a magic wand and/or thresholding tool to easily select materials with similar gray values in either one slice or the whole volume.
  c. Move a few slices ahead or backward and color in the material on this slice.
  d. Press **Ctrl** + **I** to extrapolate the labeled material in between your previously colored slices.
    **CRITICAL:** It is important to press **Ctrl** + **I** in this step. Otherwise, the extrapolating will fail.
  e. Browse through these slices to check that the labels remain close to the material.
  f. Once satisfied, press **Add** in **Selection.** This will add the painted area to your material (Figure 3D).
    **CRITICAL:** It is important to press **Add** in **Selection** before proceeding to the next step.
    ***Note:*** Check the “**current slice**” box to add the painted region on the current slice to your material.
    ***Note:*** When the marked part is not added to the material, **Add** and **Minus** in **Selection** will turn red.
    ***Optional:*** Back to **Project**, right-click raw.labels∗ object, select the option **Volume Rendering > Create** to preview the shape of target material.
  g. Label all internal structures as in Steps 22a to 22f.
    ***Note:*** The sample structure takes a long time to draw, save raw.labels∗ as data or save the entire project. Troubleshooting 4.

**Figure 3:**
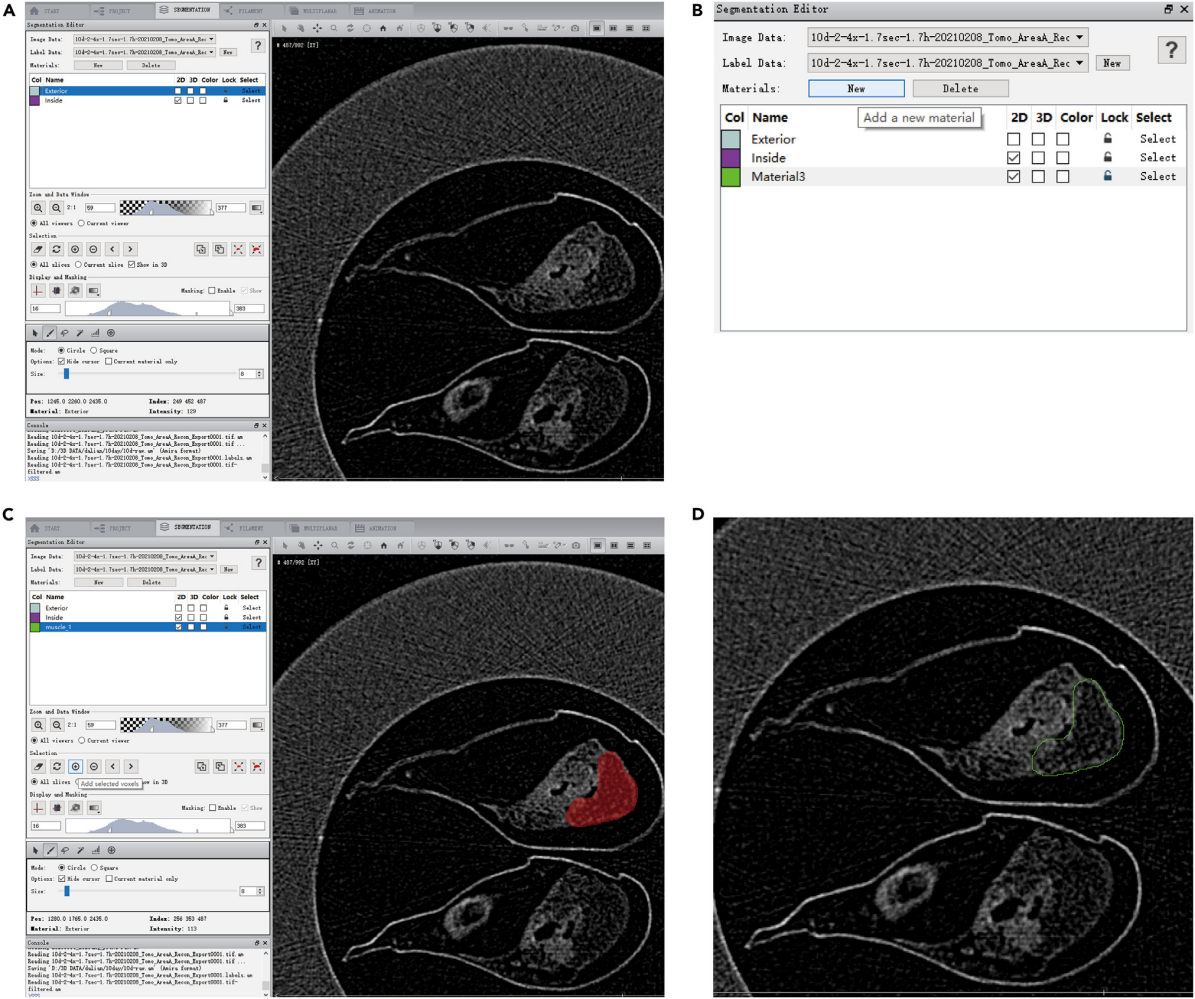
Examples of drawing sample structures (A) Main drawing interface. The left interface is toolbar and the right one is preview window. (B) Illustration of adding new material. (C) Sketch of the brush filling the desired structure. (D) Diagram of a successfully added structure in label.
23. Back to **Project**, name your labeled object internal-structures.am and click **File > Save Data.**
24. Right-click internal-structures.am, select the option **Generate Surface > Create.** Click **Apply.**
  ***Note:*** If the data size is large, a window with the title Amira: Warning will open. Press **Continue**.
  a. In **Project**, a new .surf∗ object has appeared. Right-click this object and select the option **Smooth Surface.** Set iterations value in **Parameters**, usually between 90 and 120. Click **Apply.**
  b. In **Project**, a new .smooth∗ object has appeared. Right-click this object and select the option **Surface View > Create** to preview the smoothing effect. Troubleshooting 5 and 6.
  c. Click on the .smooth∗ object and press **Simplification Editor** in **Properties.** Set faces value as the number of current faces/10 in **Simplify** and press **Simplify now.**
  d. Right-click the .smooth∗ object and select the option **Smooth Surface.** Set iterations value in **Parameters**, usually between 90 and 120. Click **Apply.**
  e. In **Project**, a new 2.smooth∗ object has appeared. Right-click this object and select the option **Surface View > Create** to preview the smoothing effect.
    ***Note:*** Remember to delete the previous Surface View object in advance.
  f. Perform iterations of the **Simplify** process and **Smooth Surface** process until objects are simple enough without loss of desired features.
  g. Click on the final object and change the object name to internal-structures-labelsFinal.smooth.surf. Click **File > Save Data.**
    ***Note:*** Do not delete previous iterations of smoothed objects until you have extracted and checked all the materials. You may find that some objects might have been smoothed too much and lost crucial details.
    **CRITICAL:** Smoothing is required to optimize 3D image.
25. Right-click raw.am object again, select the option **Edit New Label Field > Create.**
  a. Draw the exoskeleton as in Steps 22 to 23.
  b. Perform the smoothing process as in Steps 24a to 24f.
  c. Click on the final object and change object name to exoskeleton-labelsFinal.smooth.surf.
  d. Click File > Save Data.
26. Right-click exoskeleton-labelsFinal.smooth.surf object, select the option **Surface View > Create.**
  a. Click on Surface View object.
  b. In **Properties**, select the option transparent in **Draw Style** and choose the appropriate value in **Base Trans.**
27. Right-click internal-structures-labelsFinal.smooth.surf object, select the option **Surface View > Create** (Figure 4).

**Figure 4:**
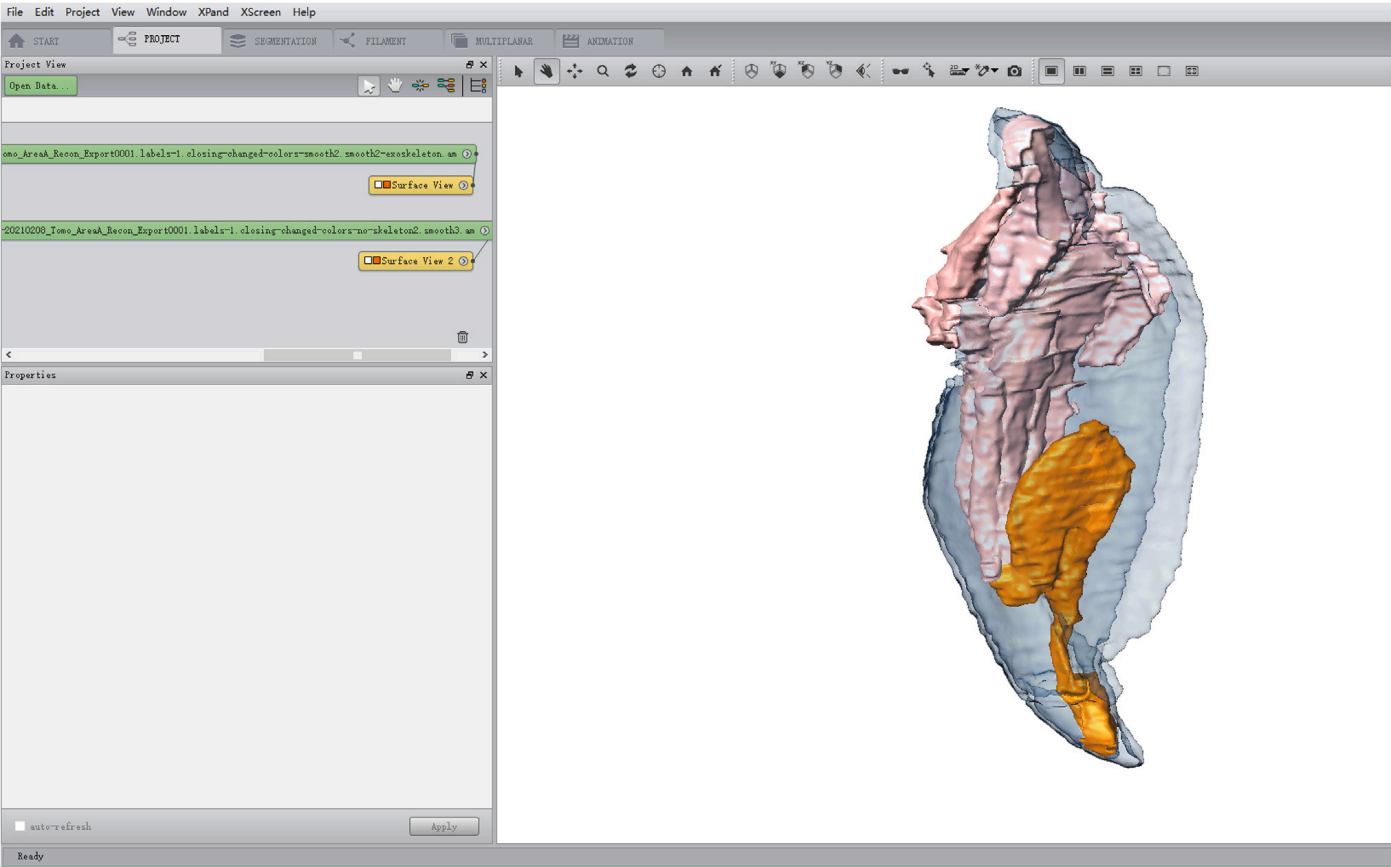
3D imaging of the final interface Main operation interface showing the finished project.
28. Click **ANIMATIONS** and a window with the title **Animation Director** will open.
  a. Right-click in the white space of **Project View** and click **Create Object.**
  b. A select box appears and select the option **Camera Orbit > Create.**
  c. In **Properties**, select z-axis in **Action.** Click the alarm symbol on the left of **Time** and select the option Time: value.
  d. Slide slider in **Animation Director** and set the next time point to e.g., 00:08 for an 8-s duration.
  e. In **Properties**, slide the slider on the right of **Time** to 360°. Click the alarm symbol on the left of **Time** and select the option Time: value.
  f. Click **Movie Creation** in **Animation Director** and a window with the title Movie Creation will open.
  g. Set **File Format** as AVI movie and choose the suitable size for your needs.
  h. Click **Create Movie.** Troubleshooting 7.

## Expected outcomes

**Figure 5:**
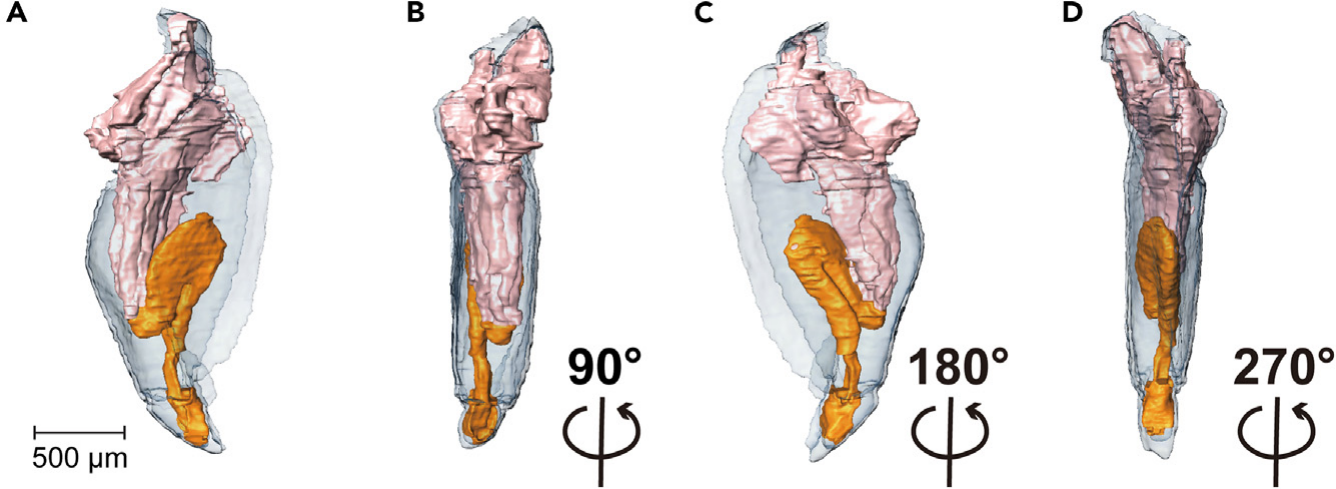
Final interactive 3D render of the regenerating leg after visualization For a Figure360 author presentation of this figure, see https://doi.org/10.1016/j.xpro.2024.103342. (A) 3D imaging of a 10 days post amputation regenerating leg. Other visualizations from three directions when rotated at 90° (B), 180° (C), and 270° (D). The exoskeleton is shown in light blue, the original internal muscle structures are shown in flesh color and the newly regenerating leg is shown in orange. Scale bar = 500 microns.

Here, a basic protocol to obtain high-resolution images of regenerating insect appendages is presented. Micro-CT scanning is a powerful and effective way to visualize regenerative structures in experimental insects. Upon completion of sample processing and imaging as above, data processing using the Amira software will allow you to generate an interactive 3D render of the desired features to be analyzed. The interactive 3D render can be viewed in several different directions (Figure 5; Supplementary Video 1). In addition, we can also use built-in algorithms to calculate surface area and volume.

## Limitations

### Spatial resolution

In scenarios requiring observation of minuscule structures or intricate anatomical features, the method may lack the necessary sensitivity for certain cellular-level investigations, despite providing a relatively high level of resolution.^3^^,^^4^ In this study, the scanner employed for generating the original images referenced was constrained by a resolution of approximately 3 μm. Nevertheless, advancements in technology and reduced costs have facilitated greater accessibility to investigate insect tissues inside opaque exoskeleton through the utilization of CT scanners capable of achieving a resolution of 0.5 μm or finer.^5^ In comparison to bench top X-ray source CT, the synchrotron source CT (e.g., APS, ALS, ESRF et al.) offer superior resolution and faster speed compared to micro-CT, achieving high magnifications and resolution to visualize tissues and even individual cells.^6^^,^^7^

### Pre-scan biological sample preparation

The low contrast sensitivity of micro-CT imaging in biological soft tissue is one of its main drawbacks.^8^ Perfusion treatment was not conducted prior to imaging the tissue structure of insect legs in this study. Enhanced imaging quality and resolution can be achieved by appropriately injecting the sample and filling the internal space with imaging media. Besides, preparing biological tissue using standard electron microscopy stains (e.g., osmium tetroxide, uranyl acetate) provide good contrast in micro-CT. Enhanced tissue contrast can also be obtained by staining the samples with heavy metal solutions, like Lugol’s iodine or phosphotungstic acid, or by using phase-contrast micro-CT (although the latter is not as readily available as conventional micro-CT scanners) to increase soft-tissue contrast.^9^^,^^10^^,^^11^

### Immobilizing the sample during scanning

In this study, cotton is employed as a tool to stabilize the sample’s position during scanning. However, the presence of cotton poses a significant challenge in image processing, potentially compromising the image’s clarity. Alternatives could be to manufacture molds from styrofoam and fix the sample between two of those molds, securing it in place. For higher density material, embedding the sample in low-concentration agar might work as well.^12^^,^^13^

### Others

To achieve a high-quality micro-CT image, it is typically necessary to subject the soft tissue sample to specific preparatory procedures like immobilization, dehydration, staining, or removing unnecessary background. These treatments may induce alterations in the sample’s structure (like tissue shrinkage) or chemical makeup, potentially influencing the precision of the imaging outcomes.^2^^,^^14^^,^^15^

## Troubleshooting

### Problem 1

Tissue shrinkage or even destruction during fixation and drying (related to steps 5 and 7).

Some of the regenerated leg tissue was damaged during sample collection. Perhaps samples were kept in fixative or formaldehyde solutions for excessive time, which has the potential to impact the specimen’s morphology or modify tissue properties. Additionally, samples were mistakenly left at room humidity after critical point drying. To ensure reliable and accurate outcomes for your experiment, consider implementing the following potential strategies.

### Potential solution

• Use sharp scissors to cut off the entire leg.
• Perform gradient dehydration as soon as possible after 24 h of fixation.
• Samples can be subjected to critical point drying, freeze-drying, or chemical drying using Hexamethyldisilazane (HMDS) to enhance tissue contrast during scanning without the presence of a liquid medium.^2^
• After critical point drying, keep samples for long-term storage in a vacuum dryer.

### Problem 2

Image blur or artifact during visualization (related to step 21).

The presence of moisture in samples may lead to image distortion or anomalies. Or slight movements or vibrations of samples happened during the imaging procedure induce image blurring or artifacts.^14^

### Potential solution

• Make sure the sample is dehydrated.
• Make sure the samples are stabilized before scanning.^16^

### Problem 3

Noise and different artefacts (related to step 21).

Quantum noise, stemming from statistical errors in low photon counts, manifests as scattered bright and dark streaks in image slices. Beyond that, additional noise can be introduced during the reconstruction process.^17^

### Potential solution

• This artifact can be mitigated by employing higher beam intensities (increased μAs) to enhance signal-to-noise ratios (SNR). Moreover, elevating source voltage and capturing more projection images can help reduce noise.
• Prolong exposure times and utilize filters can diminish artifacts in dense samples. Additionally, enhancing SNR through frame averaging is beneficial.
• Depending on the Amira license, various post-processing filters (e.g., Gaussian filter) are available to reduce noise in the reconstructed data.^18^
• CTAn, as part of the Bruker Skyscan software, has some filtering and post-processing abilities.

### Problem 4

When saving the document and opening it again, raw.labels.am object and raw.am objects are separate (related to step 22g).

Objects were saved as data separately.

### Potential solution

• Save the entire project directly.
• Right-click raw.am object, select the option **Ortho Slice.** Right-click **Ortho Slice**, select **Color Wash.** Right-click “□” in front of **Color Wash** and select the option **Data**, link it to raw.labels.am object.

### Problem 5

There are small holes in the surface during the preview (related to step 24b).

There are errors when using software to extrapolate the labeled material. Or the structure edge of the sample was not drawn exactly according to the boundary.

### Potential solution

• Fill in the parts that need to be drawn according to the sample structure.
• Right-click raw.labels.am object, select the option **Closing** to close these holes. In **Properties**, **Type** select **Ball**, **Interpretation** choose **3D** and finally enter the appropriate size. Select **Apply.**

### Problem 6

Object cannot be labeled (related to step 24b).

This object type maybe 16-bit.

### Potential solution

• Click this object, select the button **Convert Image Type Editor** in **Properties** and change **Output Type** to 8-bit.

### Problem 7

The animation is not clear (related to step 28 h).

The user has only limited control over video format settings (like compression factors, etc.) in Amira.

### Potential solution

• Set **File Format** as MPEG movie in **Movie Creation.**
• Use other software to improve animation clarity, such as Topaz Video Enhance AI.
• As an alternative, the user can also save the animated frames as individual, high-resolution PNG or uncompressed TIFF images and then use appropriate video editing software to create clearer animations using video codecs with their own preferred settings.

## Resource availability

### Lead contact

Further information and requests for resources and reagents should be directed to and will be fulfilled by the lead contact, Chonghua Ren (renchonghua111@m.scnu.edu.cn).

### Technical contact

Technical questions on executing this protocol should be directed to and will be answered by the technical contact, Chonghua Ren (renchonghua111@m.scnu.edu.cn).

### Materials availability

This study did not generate new unique reagents.

### Data and code availability

This study did not generate new datasets.

## Acknowledgments

We thank the South China National Botanical Garden for the support of critical point drying and micro-CT scanning. This work was supported by the 10.13039/100014717National Natural Science Foundation of China (grant nos. 32070500, 32370439, 32220103003, and 31930014 to C.R. and S.L.), the 10.13039/501100003453Natural Science Foundation of Guangdong Province (grant no. 2021B1515020044 to C.R.), the Department of Science and Technology in Guangdong Province (grant no. 2019B090905003 to S.L.), and the 10.13039/501100017610Shenzhen Science and Technology Program (grant no. KQTD20180411143628272 to S.L.).

## Author contributions

L.L. organized the protocol of imaging; L.L. and K.C. performed imaging operations; L.L., K.C., and S.Z. prepared the figures; J.Z. summarized the problems and limitations for this study; S.Z. sorted out the process of preparation before scanning; C.Z., Y.W., and S.L. improved the manuscript; and C.R. provided conceptual guidance for this study. All authors wrote and edited the manuscript.

## Declaration of interests

The authors declare no competing interests.

## Supplemental information

Figure360. An author presentation of Figure 5
